# Influence of stressors and possible pathways of onset of seventh graders’ suicidal ideation in urban and rural areas in Taiwan

**DOI:** 10.1186/1471-2458-13-1233

**Published:** 2013-12-27

**Authors:** Yi-Chen Chiang, Tony Szu-Hsien Lee, Lee-Lan Yen, Chi-Chen Wu, Dai-Chan Lin, Baai-Shyun Hurng, Hsing-Yi Chang

**Affiliations:** 1 School of Public Health, Chung Shan Medical University, Tai-Chung, Taiwan; 2 Department of Family and Community Medicine, Chung Shan Medical University Hospital, Tai-Chung, Taiwan; 3 Department of Health Promotion and Health Education, National Taiwan Normal University, Taipei, Taiwan; 4 Institute of Health Policy and Management, College of Public Health, National Taiwan University, Taipei, Taiwan; 5 Institute of Population Health Sciences, National Health Research Institutes, No.35, Keyan Rd., Zhunan Township, Miaoli County 350, Taiwan; 6 Health Promotion Administration, Ministry of Health and Welfare, Taichung, Taiwan

**Keywords:** Taiwan, Suicidal ideation, Adolescent, Stressor, Urban, Rural

## Abstract

**Background:**

Suicide is the second leading cause of death among young people in Taiwan. However, few studies have investigated children’s suicidal ideation, and longitudinal studies are particularly rare. The purposes of this study were: (1) to describe the proportion of students with suicidal ideation in one month and incidence of suicidal ideation in the 7th graders (the first year of junior high school) living in urban and rural areas; (2) to realize the influence of perceived stressors on the onset of 7th graders’ suicidal ideation; and (3) to explore possible pathways through which trigger factors and perceived stressors lead to suicidal ideation.

**Methods:**

A total of 1,589 students were followed from grade 4 to grade 7. Logistic regression was then used to investigate the influence of perceived stressors on the onset of suicidal ideation in grade 7. Structural equation modeling was used to analyze possible pathways through which trigger factors led to increased pressure from certain stressors which in turn resulted in suicidal ideation.

**Results:**

The proportion of students with suicidal ideation in one month was 2 to 3 times higher in both areas compared to that in elementary school. However, the incidence in the rural area showed a large increase from 10.2% and 9.5% in grades 5 and 6 to 15.5% in grade 7. Urban–rural difference was observed. Important stressors and pathways of suicidal ideation differ between urban and rural areas.

**Conclusions:**

This study showed that the influential stressors in urban and rural areas might be different. Thus, interventions focused on coping skills for regional specific stressors and trigger factors could be beneficial in the transition time.

## Background

Suicide is a major public health problem worldwide and is one of the main causes of death and health burden in most developed and many developing countries. In Taiwan, suicide has been the ninth leading cause of death throughout 1999–2009 [1]. The annual suicide prevalence rate in Taiwan has been greater than 13 per 100,000 since 2002 [1], which indicates a high prevalence according to WHO criteria [2]. In Taiwan, greater attention should be paid to the problem of suicide in young people, particularly as youth suicide is second only to motor vehicle traffic accidents as a cause of death in the population aged 15–24 years. The suicide prevalence rate in young people in 2003 and 2004 was 3.2 and 3.5 per 100,000 respectively, with a 9% annual increase in 2004 [1]. Suicide is among the 8th to 10th leading causes of death in those aged 5–14 years [3]. However, as suicide is rarely reported in children under 14 years of age, suicide in this age group is likely to be underestimated [4].

The progression and severity of suicide behaviors range from suicidal ideation to attempted suicide and completed suicide [5-7]. Much research has found that suicidal ideation is a significant risk factor for both suicide attempts and committing suicide [8-10]. In Taiwan, a study [11] found that 51.6% of adolescents in urban junior high schools were at high-risk of suicide and needed further assessment for at-risk suicidal ideation. Our previous analysis of the CABLE study [12] found that the prevalence of ever having suicidal ideation among 4th graders was 19.77%. Another Taiwanese qualitative study found that participants experienced suicidal ideation, trigger events and situations that made them contemplate ending their own lives when they were in elementary school or junior high school [6]. This indicated that the risk of suicide is high in Taiwan, even in younger age groups.

There is a lack of research on factors associated with suicidal ideation in Taiwanese children, and school-based surveys focusing on nonclinical samples are particularly rare. As grade 7 is the first year of junior high school in Taiwan, this is an important transition time (as they progress from elementary school to junior high school) during which children may face a variety of stressors (such as academic pressure, new interpersonal relationships) and changes (such as body shape). During the transition period from childhood to adolescence, these individual, family and peer factors are the most important sources of “risk” and “support” [13]. On the other hand, according to the “Transactional model of stress and coping” [14], when someone feels that a certain stressor may threaten or challenge his/her life, he/she will make cognitive appraisals of the significance of the stressor. Therefore, if people perceive high pressure from stressors, but do not have the appropriate coping skills or resources to deal with these pressures, they may develop negative affect as a consequence (e.g., suicidal ideation and depressed mood). In addition, previous studies have pointed to different prevalence rates of suicidal behaviors between urban and rural areas. However, extensive research in this area has rarely been conducted in Asian countries. Thus, the purposes of this study were: (1) to describe the proportion of students with suicidal ideation in one month and incidence of children’s suicidal ideation in grade 7 with a focus on regional differences; (2) to realize the influence of perceived stressors on the onset of 7th graders’ suicidal ideation in the two areas; and (3) to explore possible pathways through which trigger factors result in high perceived pressure from stressors leading to suicidal ideation in 7th graders.

## Methods

### Participants

Data came from the Child and Adolescent Behaviors in Long-term Evolution (CABLE) Project, initiated in 2001. CABLE was funded by the National Health Research Institutes (NHRI) in Taiwan and was approved by the Institutional Review Board NHRI. The study was designed to observe the development of children based on the ecological model, which emphasized that different levels including individual, interpersonal, organizational, community and public policy shape the development of a child. The CABLE study randomly selected 18 public elementary schools from Taipei city (representing an urban area) and Hsinchu county (representing a rural area) in Taiwan. As there are only a few private primary schools in these two areas (10 from 152 primary schools in Taipei City and 1 from 79 primary schools in Hsinchu County), and as the origin and family background of the students in these schools in quite dissimilar to the students in public schools, these private schools were excluded from the sample population. Based on the number of 1st grade students, the schools were categorized as small (50–199 students), medium (200–399 students) or large (more than 400 students). To ensure that the number of children chosen from each type of school was approximately equal, it was determined to select six small schools, two medium schools and one large school from each location. In each school, all of the students in grades one and four (referred to as cohorts 1 and 2, respectively) and their parents were selected as the sample. Further details about the sampling procedure for the CABLE study have been described elsewhere [15]. Informed consent was obtained from each child’s parents before the baseline survey in 2001. Each student whose parents agreed to their participation filled out a self-completed questionnaire under the direction of research assistants in the classroom during school hours. The entire survey was completed in 40 minutes.

The CABLE cohort 2 consisted of 2,075 fourth graders who completed questionnaires at baseline in 2001. A total of 1,593 students from 18 representative elementary schools were followed from grade 4 (in 2001, ages ranged from 9 to 10 years) through grade 7 (in 2004, ages ranged from 12 to 13 years). Four students were excluded due to missing data for the question on suicidal ideation in one or more years. As a result, a total of 1,589 participants were included in the study, giving a follow-up rate of 76.6%. There were 809 boys (50.9%) and 780 girls (49.1%), and 812 (51.1%) children from Taipei city and 777 (48.9%) from Hsinchu county. Losses to follow up were due to absence from school due to illness, refusal to participate, and overseas travel.

### Measures

The dependent variable in our analyses was the onset of children’s suicidal ideation during the transition time (grade 7). Suicidal ideation was measured every year from grade 4 through grade 7 by asking participants “Did you ever think of ending your own life? (not wanting to live or wanting to die)” There were 5 possible responses: “never,” “yes, but not during the past month,” “once or twice during the past month,” “many times during the past month,” and “almost every day during the past month.” The four-year longitudinal data was used to estimate the proportion of students with suicidal ideation in one month and the incidence of children’s suicidal ideation. The CABLE survey was conducted during October and November every year, and September is the first month of the academic year in Taiwan. Therefore, students who reported having never had suicidal ideation prior to the first month of grade 7 who then reported suicidal ideation for the first time in grade 7 were considered as new cases. Students who had had suicidal ideation during elementary school were excluded in the analysis of risk factors for incident suicidal ideation.

**Figure 1:**
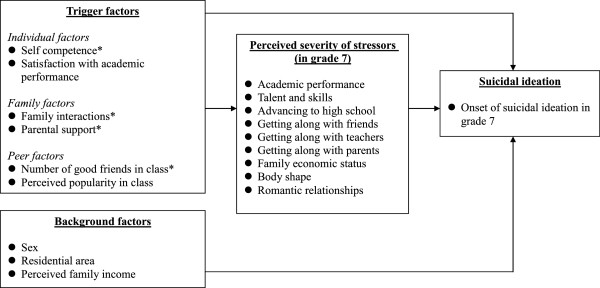
**Possible pathway for onset of suicidal ideation in grade 7.** *: The variable was examined in two ways: (1) the value in grade 7; and (2) the difference between the values in grade 6 and grade 7.

Figure 1 shows our research framework for possible pathways of onset of suicidal ideation in grade 7. We hypothesized that trigger factors including individual, family level, and peer factors had effects on the severity of perceived stressors, which in turn had effect on the onset of suicidal ideation. Because some independent variables were measured when participants were in grade 6 and in grade 7, we investigated the effects of these variables in two separate ways: (1) the value in grade 7 (in order to investigate immediate effects); and (2) the difference between the value in grade 6 and grade 7 (in order to examine the effect of change in these variables on suicidal ideation). About the definition of explanatory variables were displayed in Additional file 1.

### Data analysis

We first used latent growth curve modeling to test the linear and quadratic increase of the proportion of students with suicidal ideation in one month. The chi-square test was used to compare the incidence of suicidal ideation in 7th graders by sex and urban–rural location. Logistic regression was then used to investigate the influence of perceived stressors on the onset of suicidal ideation in grade 7. Structural equation modeling was used to analyze possible pathways through which trigger factors led to increased pressure from certain stressors which in turn resulted in suicidal ideation. As some variables were ordinal, we calculated a polychoric correlations matrix and asymptotic covariance matrix as the input data and used the weighted least squares estimation method to estimate the parameters [16]. Assessment of data-model fit was based on the following criteria drawn from the literature [16-18]: (1) the Root Mean Squared Error of Approximation (RMSEA): an absolute fit index which should be below 0.08; (2) the Goodness-of-Fit Index (GFI) and the Adjusted Goodness-of-Fit Index (AGFI): both should be above 0.9; and (3) χ^2^/df: which should be less than 5. Analyses were carried out using the SAS statistical software package (9.2 version), Mplus (5.2 version), and LISREL program (8.8 version).

## Results

**Figure 2:**
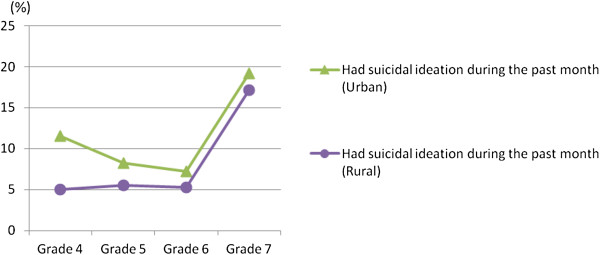
Proportion of students with suicidal ideation in one month from grade 4 to grade 7 in urban and rural areas.

The proportion of 7th graders with suicidal ideation in one month is presented in Figure 2. The proportion of children’s suicidal ideation between grade 4 and grade 6 (elementary school) ranged from 7.3% to 11.6% in the urban area, whereas the proportion ranged from 5.0% to 5.5% in the rural area. The proportion of students with suicidal ideation in one month was highest in grade 7 when the children entered junior high school (urban area: 19.2%; rural area: 17.1%), which is an increase of twofold to threefold compared to that observed during elementary school years. According to the result of latent growth curve modeling, the first order sample proportion in grade 7 was higher than the proportion between grade 4 and grade 6. Also, the estimate of linear growth was −1.035 (*p* < 0.001) and the estimate of quadratic growth was 0.545 (*p* < 0.001). It means there was rapid growth of students’ suicidal ideation especially in grade 7.

**Figure 3:**
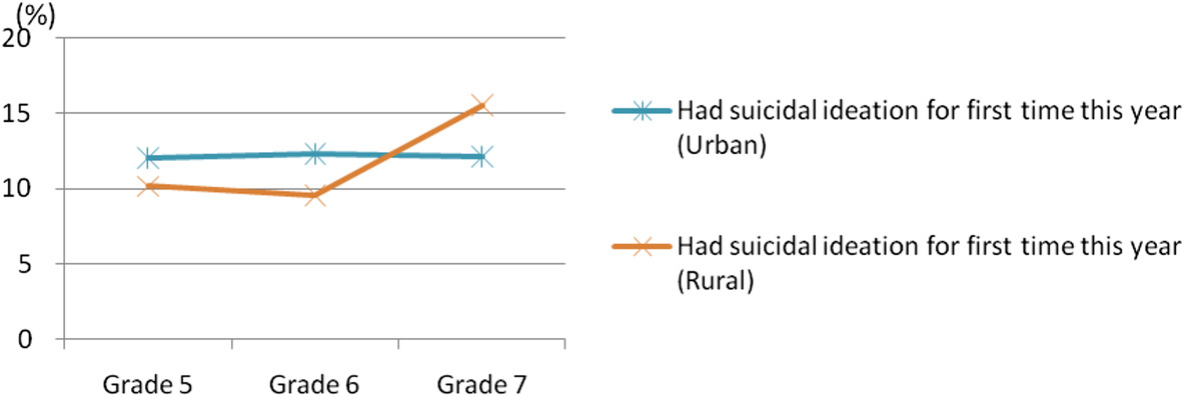
Incidence of children’s suicidal ideation from grade 5 to grade 7 in urban and rural areas.

Figure 3 shows the incidence of children’s suicidal ideation from grade 4 to grade 7 in urban and rural areas. The incidence of suicidal ideation was fairly stable in the urban area. However, the incidence in the rural area showed a large increase from 10.2% and 9.5% in grades 5 and 6 to 15.5% in grade 7. In addition, the incidence in the rural area (15.5%) in grade 7 was higher than that observed in the urban area (12.1%) in the same year.

Table 1 shows the new case rate of suicidal ideation in 7th graders. The new case rate in Hsinchu County (rural area) was 11.2% which was significantly higher than that of 6.9% observed in Taipei City (urban area) (χ^2^ = 8.97, *p* < 0.01). However, there was no statistically significant sex difference.

**Table 1:** **Sex differences and urban-rural disparity in the new case rate of suicidal ideation in 7**^**th **^**graders**

	**Taipei city (Urban area)**			**Hsinchu county (Rural area)**			**Total**		
	**Boys**	**Girls**	**Subtotal**	**Boys**	**Girls**	**Subtotal**	**Boys**	**Girls**	**Subtotal**
	**(n=412)**	**(n=400)**	**(n=812)**	**(n=397)**	**(n=380)**	**(n=777)**	**(n=809)**	**(n=780)**	**(n=1589)**
New case rate (%)	6.8	7.0	6.9	10.6	11.8	11.2	8.7	9.4	9.0
Sex difference	χ^2^ = 0.01			χ^2^ = 0.31			χ^2^ = 0.24		
Urban-rural disparity	χ^2^ = 8.97**								

χ^2^: The Chi-Square test.

**: P < 0.01.

**Figure 4:**
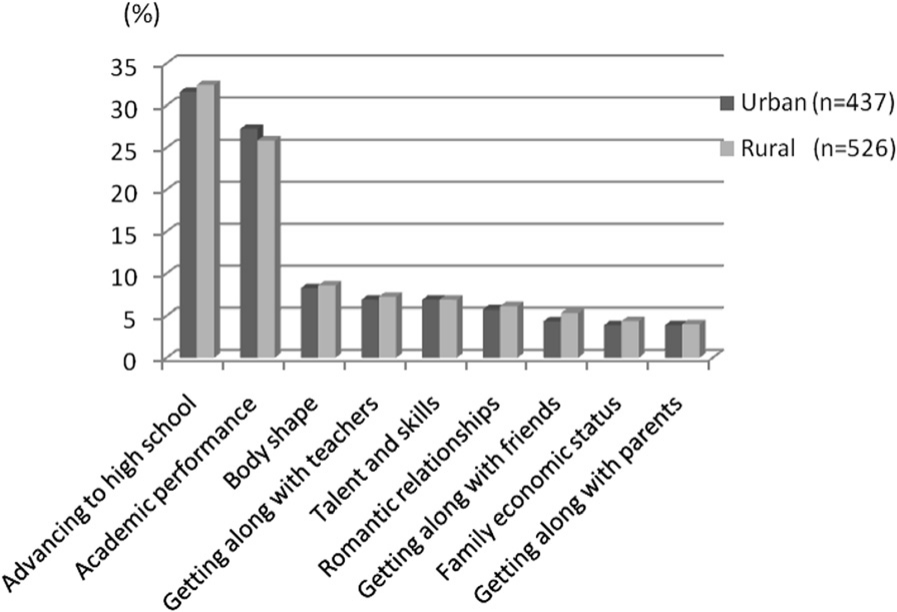
The proportion of students perceiving a high degree of pressure from stressors in grade 7 in urban and rural areas.

The proportion of students in urban and rural areas who perceived high levels of stressors in grade 7 and the relative magnitude of these perceived stressors are presented in Figure 4. Advancing to high school and academic performance were the top two stressors for 7th graders in both urban and rural areas. More than 30% of 7th graders reported that they felt a high degree of stress from worrying about the entrance exam for entering senior high school in three years time (grades 10–12 in Taiwan), even during the first few months of grade 7. Moreover, 27.2% and 25.9% of urban and rural youth, respectively, indicated that they felt highly pressured about their academic performance at school. Furthermore, there were no statistically significant differences in the proportion of students perceiving high levels of each stressor or the relative magnitude of these stressors in grade 7 between two areas.

Table 2 shows the results from the logistic regression analysis of the effects of perceived stressors on the onset of suicidal ideation in grade 7. After controlling for sex and perceived family income, we found that a higher total score for the nine perceived stressors was significantly associated with suicidal ideation in 7th graders in both urban and rural areas (OR = 1.12 and 1.13, respectively, in Model 1). Moreover, the model 2 showed that different stressors were significantly associated with suicidal ideation in 7th graders in two areas. In the urban area, 7th graders who felt highly pressured about their body shape and getting along with friends were more likely to think about suicide for the first time in grade 7 than those who did not feel pressured by these two stressors (OR = 4.86 and 5.91, respectively). However, in the rural area, academic performance and getting along with parents were the two variables most strongly associated with suicidal ideation in grade 7 (OR = 2.64 and 4.01, respectively). Collinearity between independent variables in Model 2 was not substantial as the VIF values among all covariates were much less than the recommended cut point of 10.

**Table 2:** **Logistic regression models for onset of suicidal ideation in 7**^**th **^**graders**

	**Urban (n=434)**^**a**^			**Rural (n=512)**^**a**^		
**Degree of pressure from stressors**	**OR**	**(95% C.I.)**	**p**	**OR**	**(95% C.I.)**	**p**
***Model 1***^***b***^						
Total score	**1.12**	**(1.06,1.17)**	***	**1.13**	**(1.08,1.18)**	***
***Model 2***^***b***^						
Advancing to high school	0.67	(0.29,1.59)	NS	1.02	(0.47,2.22)	NS
Academic performance	2.13	(0.96,4.71)	NS	**2.64**	**(1.24,5.65)**	*
Body shape	**4.86**	**(1.96,12.03)**	***	0.74	(0.25,2.19)	NS
Getting along with teachers	0.90	(0.25,3.22)	NS	1.58	(0.58,4.33)	NS
Talent and skills	1.73	(0.58,5.14)	NS	1.92	(0.73,5.05)	NS
Romantic relationships	1.32	(0.36,4.83)	NS	0.62	(0.17,2.30)	NS
Getting along with friends	**5.91**	**(1.86,18.73)**	**	1.73	(0.58,5.16)	NS
Family economic status	1.93	(0.46,8.00)	NS	1.25	(0.34,4.61)	NS
Getting along with parents	0.21	(0.03,1.64)	NS	**4.01**	**(1.25,12.81)**	*
***Goodness-of-fit indices***						
**(1) C**	Model 1: 0.71			Model 1: 0.71		
	Model 2: 0.69			Model 2: 0.71		
**(2) Hosmer and Lemeshow test**	Model 1: **χ**^2^=3.38 (df=8, p=NS)			Model 1: **χ**^2^=5.38 (df=8, p=NS)		
	Model 2: **χ**^2^=8.08 (df=8, p=NS)			Model 2: **χ**^2^=4.37 (df=7, p=NS)		

Note: ^a^Background factors including sex and perceived family income were controlled in both Model 1 and Model 2. Neither sex nor perceived family income had a significant association with the dependent variable. ^b^The total score for the 9 stressors (ranging from 9 to 45) was used in Model 1. Two categories, high-pressured (participants who answered “greatly” or “very greatly” in response to questions about the degree of pressure from stressors) and low-pressured (participants who did not answer “greatly” or “very greatly”) were used in Model 2. C is one of the goodness-of-fit indices, which is the comparison between the predicted probabilities and the observed responses. It is provided by SAS.

*: P < 0.05; **: P < 0.01; ***: P < 0.001; NS: non-significant.

**Figure 5:**
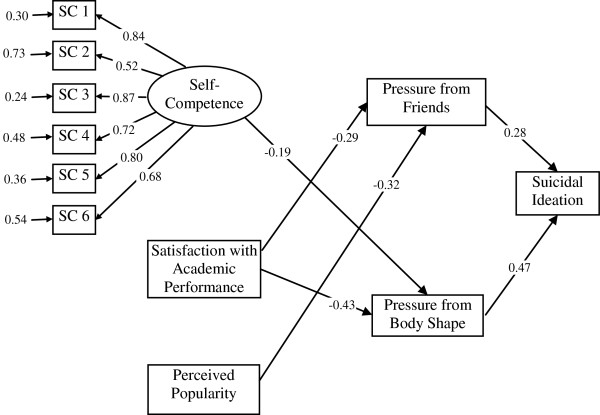
**The standardized coefficients of associations in the possible pathway of onset of suicidal ideation in grade 7 in the urban area.** (Note: Every line in the figure denotes a statistically significant relationship. Correlation between each two exogenous variables were controlled in this model).

Figure 5 shows the possible pathway of onset of suicidal ideation in 7th grade urban students using structural equation modeling. All goodness-of-fit indices indicated that the model fitted the urban data well (χ^2^/df =132.73/40 = 3.32, RMSEA = 0.074, GFI = 0.98, and AGFI = 0.96). We found that if 7th graders in the urban area perceived that they were less popular in class or were less satisfied with their academic performance, they were more likely to experience high pressure from getting along with friends, which in turn was associated with onset of suicidal ideation in grade 7. In addition, urban students who had lower perceived self-competence or less satisfied with their academic performance in grade 7, were more likely to experience high pressures from body shape, which in turn was associated with onset of suicidal ideation in grade 7.

**Figure 6:**
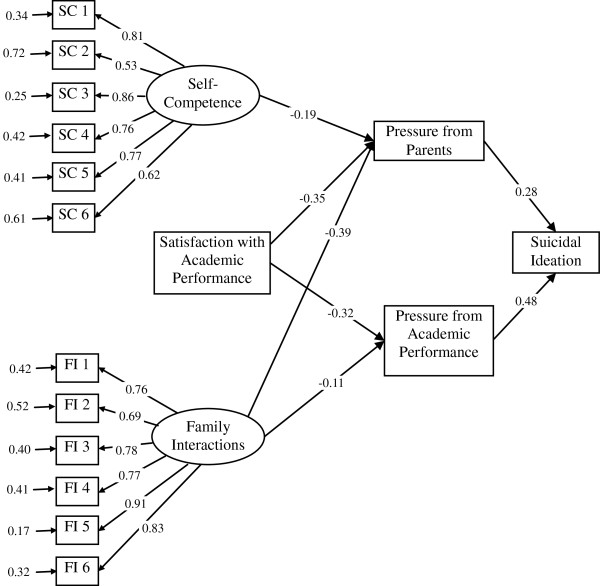
**The standardized coefficients of associations in the possible pathway of onset of suicidal ideation in grade 7 in the rural area.** (Note: Every line in the figure denotes a statistically significant relationship. Correlation between each two exogenous variables were controlled in this model).

The possible pathway of onset of suicidal ideation in grade 7 for rural students is shown in Figure 6. All goodness-of-fit indices indicated that the model fitted the rural data well (χ^2^/df = 290.49/98 = 2.96, RMSEA = 0.063, GFI = 0.97, and AGFI = 0.96). We found that 7th graders in the rural area who had lower perceived self-competence, fewer family interactions, and were less satisfied with their academic performance, were more likely to experience high pressure from getting along with their parents, which in turn was associated with onset of suicidal ideation in grade 7. In addition, students who perceived fewer family interactions and were less satisfied with their academic performance, were more likely to experience high pressure from academic performance, which in turn was associated with onset of suicidal ideation in grade 7.

## Discussion

This study examined the incidence of suicidal ideation among 7th graders using a cohort. We observed urban–rural difference. An urban–rural disparity in children’s suicidal ideation has been previously reported in young people in China [19-22], however, the direction of this disparity has been inconsistent between studies. Inconsistent conclusions about the direction of urban–rural disparities in children’s suicidal ideation may have resulted from comparisons of results from different cross-sectional surveys (with possible differences in study design), rather than a comparison of rates between areas in the one study [4]. The results of the present study that has used longitudinal data to capture changes in children’s suicidal ideation over time in urban and rural areas are therefore extremely valuable.

It has already been shown that poor academic achievement is an important risk factor for suicidal ideation among Chinese adolescents [20]. This reflects the fact that education and success in exams are regarded as the primary pathway of upward mobility in contemporary Chinese society. At school, students are taught that hard work and high educational achievement are important forms of self-improvement [20]. Fear of failure and actual failure on exams has been shown to lead to anxiety and depression in Chinese populations [23,24]. Our findings provide further evidence that academic performance is an important stressor among 7th graders. Although many 7th graders felt highly stressed about their academic performance in both urban and rural areas, only rural students experienced high pressure from academic performance that was associated with onset of suicidal ideation in grade 7. One explanation for this difference could be that the educational resources available in Taiwan’s rural areas are less plentiful than in urban areas. The geographical size of Hsinchu County (the rural area) is 5.25 times greater than that of Taipei City [25,26]. However the number of schools in Hsinchu County is about half that of Taipei City [27]. Moreover, the student-teacher ratios in rural areas (ratios of 18.17, 17.55, and 17.61 for elementary schools in 2001 ~ 2003, respectively; a ratio of 15.28 for junior high schools in 2004) are higher than those in urban areas (ratios of 16.40, 16.05, and 16.10 for elementary schools in 2001 ~ 2003, respectively; a ratio of 13.84 for junior high schools in 2004) both in elementary schools and junior high schools [27], indicating a potentially lower quality of education in rural areas. Another explanation could be that children living in urban areas have already faced high pressure for academic achievement during elementary school. Grade 7 is the first year of junior high school in Taiwan and students need to take the senior high school entrance exam before the 10th grade. As a result 7th graders are under pressure to study much harder than when they were in elementary school. This sudden increased strain to excel academically combined with deficient educational resources could lead rural students who have never had suicidal ideation during elementary school years to consider suicide in grade 7.

Body-image dissatisfaction is an important contributor to suicidal ideation in adolescents that has been found in previous research [28]. However, differences in the influence of body image on children’s suicidal ideation between urban and rural areas are still unclear. The present study found that if an urban student perceived a high degree of pressure about his or her body shape, he or she was more likely to experience suicidal ideation in grade 7. This finding could be due to more frequent exposure to physical comparisons among adolescents in urban areas. In addition, Szabo and Allwood [29] found that body-figure preferences differed between urban and rural areas. Most urban adolescents have a desire to be smaller. However, data from rural areas suggests milieu-specific factors in this regard, with fewer respondents desiring to be smaller. This could influence the emergence of eating disorders and preference for thinness in urban areas. As a result, although the proportion and relative magnitude of pressure concerning body shape was similar between urban and rural areas in our study, we found that the onset of suicidal ideation in grade 7 was associated with body shape-related pressure only in urban students.

A previous study found that poor parental relationships were more strongly associated with suicidal ideation than peer relationships among children aged 12 to 13 years [30]. However, another study found that greater levels of perceived peer rejection and lower levels of close friendship support were directly associated with more severe suicidal ideation in adolescent inpatients [31]. Therefore, research findings have been inconsistent regarding the effect of peer and parent relationships on children’s suicidal ideation. The findings from our study extend the current literature. We found that getting along with friends was significantly associated with suicidal ideation in urban students, whereas getting along with parents was significantly associated with suicidal ideation in rural students. A possible explanation is that urban students may enter a period in which they pay more attention to their peers than their families, earlier than rural students. An alternative explanation might be that adolescents in rural areas start looking for independence from their parents earlier, whereas adolescents in urban areas are more concerned with fitting in to their peer group. Differences in parenting between urban and rural areas could also make a contribution, as parents in urban areas are more likely than those in rural areas to pay attention to the privacy concerns of their adolescent children and adjust their parenting strategies accordingly [32].

A previous study has found that chronically stressful social and family situations (such as parental disharmony) and acute life stresses (such as changing schools) increase the likelihood of reporting unexplained physical symptoms in children and adolescents [33]. In the present study, we examined the effect of trigger factors on perceived severity of stressors and onset of suicidal ideation by looking at both immediate effects and effects of changes to trigger factors. We found that the immediate effects of trigger factors were more pronounced.

We found that low self-competence and less satisfaction with academic performance acted as trigger factors in both urban and rural students. This indicates that individual level risk factors are important for adolescents, particularly for the adaptation required during the transition period from elementary school to junior high school. However, perceived popularity was an additional significant trigger factor in urban youth, and family interactions was an additional significant trigger factor in rural youth. Therefore, it appears that peer level risk factors are important for urban students, whereas family level risk factors are important for rural students during this period.

Children and adolescents who have low perceived self-competence are more likely to report high levels of physical symptoms [33]. Our study further discovered that low self-competence had significant effects in both urban and rural students, however, the pathways through which low self-competence was related to suicidal ideation were different. Low perceived self-competence in urban students was associated with perceived high pressure from body shape, which in turn was associated with suicidal ideation. In rural students on the other hand, low self-competence was associated with perceived high pressure from getting along with parents, which in turn was associated with suicidal ideation. The potential neighborhood and contextual influences that result in different pathways of suicidal ideation in adolescence deserve further research.

This study has a couple of limitations. First, given the secondary data analysis nature of this study, we could only use single item of screening the suicidal ideation in school children. That was an important limitation. However, the measure was adopted from the US youth risk behavior survey (YRBS) [34]. This single item has been widely used in American and European young students (European School Survey Project on alcohol and other drugs) [35], in the WHO international survey for school-aged children (Health behavior in school-aged children study, HBSC) [36], in the Korea youth (Korea Youth Risk Behavior Web-based Survey, KYRBWS) [37], in Mexico city [38], and in Chinese adolescents in Taiwan [39-41]. We believed this single item had its value even though it was not perfect. The longitudinal nature of our data enabled the estimation of the incidence of suicidal ideation and factors associated with onset of suicidal ideation.

Second, we were unable to capture the severity of perceived stressors and some trigger factors during elementary school years. The 7th grade is an important transition period for young people in Taiwan as it is the first year of junior high school and therefore signifies the important transition from elementary school to high school. Therefore, participants were particularly asked about the stressors they faced when entering grade 7 in the CABLE project and many of these variables were not measured in earlier waves of follow-up.

A third limitation was that only 2 locations were used to represent urban and rural areas due to concerns about follow-up and limited funding. Results should be generalized with caution. However, as the CABLE project is a cohort study in a general population group, in spite of only tracing one urban and one rural area, the major purpose and contribution is to find the different patterns or correlates of such behaviors and to explore the potential urban–rural disparity. It may help us to establish the model or preventive strategy specifically applying to urban and rural areas.

## Conclusions

In conclusion, greater attention needs to be paid to the onset of suicidal ideation in 7th graders. Children’s emotional status and presence of suicidal ideation should be a focus of research and interventions, to prevent increases in suicidal ideation in children and adolescents. The influence of perceived stressors on the onset of suicidal ideation in 7th graders may differ between urban and rural areas, and parents, teachers, and professionals in urban and rural areas should be aware of children’s perceived strain from specific stressors. Furthermore, tailored interventions focused on mastering coping skills for specific stressors and trigger factors that are important for the different areas could be beneficial, especially in the transition period from elementary school to junior high school. Policies aimed at providing equal educational resources to all students regardless of location should also be seriously considered. On the other hand, suicide ideation might not result in actual suicide. Studies are needed to clarify the path from suicide ideation to actual suicide.

## Consent

Written informed consent was obtained from the individual’s guardian/parent/next of kin for the participation of the survey, analysis of the data, and reporting the results in groups.

## Competing interests

The authors declare that they have no competing interests.

## Authors’ contributions

YCC participated in the design, conducted the statistical analyses, interpreted the data, and drafted the manuscript. HYC supervised the study, assisted in data interpretation, and critically reviewed the manuscript several times. TSHL and YLL helped in conducting the study and revising the manuscript. CCW and DCL helped managing and analyzing the data. HBS was in charge of fieldwork. All authors read and approved the final manuscript.

## Pre-publication history

The pre-publication history for this paper can be accessed here:

http://www.biomedcentral.com/1471-2458/13/1233/prepub

## Supplementary Material

Additional file 1 Definition of explanatory variables. Click here for file
