# Supplementary material for: Influence of stressors and possible pathways of onset of seventh graders’ suicidal ideation in urban and rural areas in Taiwan
Source: BMC Public Health. 2013 Dec 27;13:1233. doi: 10.1186/1471-2458-13-1233 (PMC3883481; doi:10.1186/1471-2458-13-1233)
Supplement: Additional file 1 — Definition of explanatory variables. [file 1471-2458-13-1233-S1.docx]

**Additional file 1.** Definition of explanatory variables

| Variable | Question | Choice | Scales/re-grouping |
| --- | --- | --- | --- |
| **Perceived severity of stressors (9 items)** | | | |
| 1. Academic performance | Do you feel stressed about this stressor? | A 5-point scale that ranged from “not at all” to “very greatly.” | 1. The total score for the 9 stressors (ranging from 9 to 45) was used to represent the overall degree of perceived pressure in grade 7.  2. Grouped as highly-pressured /low-pressured. |
| 1. Talent and skills |  |  |  |
| 1. Advancing to high school |  |  |  |
| 1. Getting along with friends |  |  |  |
| 1. Getting along with teachers |  |  |  |
| 1. Getting along with parents |  |  |  |
| 1. Family economic status |  |  |  |
| 1. Body shape |  |  |  |
| 1. Romantic relationships |  |  |  |
| **Trigger factors** |  |  |  |
| <Individual level> |  |  |  |
| 1. Self-competence **(6 items)** | (1) Do you feel you are an optimistic person? | A 5-point scale ranging from “never” to “always.” | The total score (ranging from 6 to 30) was used to represent the overall degree of self-competence in grade 6 and grade 7. |
|  | (2) Do you like to try new things? |  |  |
|  | (3) In general, do you feel you are a happy person? |  |  |
|  | (4) Do you think you are as good as others? |  |  |
|  | (5) Do you always try hard at everything? |  |  |
|  | (6) When facing problems, do you try to solve them by yourself first? |  |  |

**Appendix 1. (Cont')** Definition of explanatory variables

| Variables | Questions | Responses | Scales/regroup |
| --- | --- | --- | --- |
| 1. Satisfaction with academic performance | Are you satisfied with your academic performance? | A 5-point scale ranging from “not satisfied at all” to “very satisfied.” |  |
| < Family level> |  |  |  |
| 1. Family interactions **(6 items)** | Do you do these six daily activities with your father or mother in the past week?  (1) Chatting  (2) Having a meal together every day  (3) Doing housework together  (4) Helping with homework  (5) Playing together at home  (6) Playing together outside | A 4-point scale ranging from “never” to “almost every day”. | The total score (ranging from 4 to 24) was used to represent the overall degree of family interactions in grade 6 and grade 7. |
| 1. Parental support **(6 items)** | (1) When you face problems, do your parents encourage you? | A 4-point scale ranging from “never” to “almost every day”. | The total score (ranging from 4 to 24) was used to represent the overall degree of parental support in grade 6 and grade 7. |
|  | (2) Do your parents praise you when you are good? |  |  |
|  | (3) Do your parents comfort you when you are down? |  |  |
|  | (4) Do your parents take care of you when you don’t feel well? |  |  |
|  | (5) Do your parents listen to you? |  |  |
|  | (6) Do your parents take an interest in your school life? |  |  |
| < Peer level> |  |  |  |
| 1. Number of good friends in class | How many good friends in your class do you have? | Number |  |
| 1. Perceived popularity in class | Do you think you are a popular person in your class? | A 5-point scale ranging from “very unpopular” to “very popular.” |  |
